# Grave's Disease with Severe Hepatic Dysfunction: A Diagnostic and Therapeutic Challenge

**DOI:** 10.1155/2014/790458

**Published:** 2014-09-15

**Authors:** Ashok Krishna Bhuyan, Dipti Sarma, Uma Kaimal Saikia, Bipul Kumar Choudhury

**Affiliations:** Gauhati Medical College, Guwahati 781032, India

## Abstract

Hepatic dysfunction in a patient with thyrotoxicosis may result from hyperthyroidism per se, as a side effect of antithyroid drugs, and causes unrelated to hyperthyroidism which sometimes causes diagnostic and therapeutic difficulties. A young female patient was admitted to our hospital with symptoms of thyrotoxicosis, diffuse goiter and ophthalmopathy along with cholestatic pattern of jaundice, and proximal muscle weakness. She was treated with propylthiouracil with gradual recovery. She was continuing her antithyroid medication with regular follow-up. The patient was readmitted a few months later with worsening thyrotoxicosis, proximal muscle weakness, fever, and a hepatocellular pattern of jaundice with sepsis. Propylthiouracil was stopped and lithium along with steroid coverage was given to control her thyrotoxicosis which was later changed to methimazole. Broad spectrum antibiotic therapy was also started but without any response. During her hospital stay, the patient also developed a flaccid paraplegia resembling Guillain-Barre syndrome. IV steroid was started for the neuropathy but meanwhile the patient succumbed to her illness. So in centers where facility for radioiodine therapy is not readily available, some definite well-tested protocols should be formulated to address such common but complicated clinical situations.

## 1. Introduction

Hepatic dysfunction in patients with thyrotoxicosis may be caused by hyperthyroidism per se, as a side effect of drugs used to treat hyperthyroidism and associated rare conditions like autoimmune hepatitis or causes unrelated to hyperthyroidism like viral hepatitis, alcohol abuse, sepsis, cholangitis, or certain medications. Uncomplicated hyperthyroidism causing severe hepatic dysfunction is, however, rare. It was first reported by Habershon in 1874 [1]. The use of the antithyroid medications, especially thionamide group of drugs (propylthiouracil, methimazole, and carbimazole), in a hyperthyroid patient has been reported to cause hepatic injury. The nature of liver injury associated with the use of antithyroid drugs is drug specific, with propylthiouracil predominantly causing toxic hepatitis and methimazole or carbimazole predominantly causing a cholestatic pattern [2]. This type of liver dysfunction generally subsides after withdrawal of the offending drug. However, sometimes the presence of severe hepatic dysfunction of multifactorial origin in a patient with hyperthyroidism may complicate the clinical picture leading to difficulties in diagnosis and treatment. In addition to this, the concurrent use of antithyroid drugs in such patients may exacerbate the liver injury and may further add to the diagnostic and therapeutic dilemma. We report a case of Grave's disease presenting with severe hepatic dysfunction, sepsis, and flaccid quadriplegia which caused great diagnostic and therapeutic challenge ultimately leading to death of the patient.

## 2. Case Report

A 26-year-old female patient presented with symptoms of thyrotoxicosis in the form of palpitation, progressive weight loss in spite of increased appetite, hyperdefecation, and anxiety along with neck swelling and bulging of her eye balls for last 2 years. She also developed jaundice for last 2 months and proximal muscle weakness for last 15 days. Physical examination revealed a BMI of 15.79 kg/m^2^, resting tachycardia, a wide pulse pressure, and warm and moist extremities with diffuse thyromegaly and ophthalmopathy suggesting Grave's disease. Other significant physical findings were icterus, mild hepatomegaly with normal jugular venous pressure, and a predominantly proximal muscle weakness with preservation of the deep tendon reflexes. Laboratory investigations revealed significant suppression of thyroid stimulating hormone (TSH) (0.056 IU/L) and high free serum T4 (3.45 ng/dL) and serum T3 (4.56 nmol/L). Liver function tests revealed total bilirubin levels of 15.8 mg/dL with a predominantly conjugated fraction (12.6 mg/dL), an abnormal alkaline phosphatase of 364 U/L, and an AST and ALT of 46 U/L and 43 U/L, respectively, suggestive of a cholestatic pattern. Coagulation studies were within normal limits. Screening for routine viral markers was all negative. She was also found to have hypokalemia which was thought to be due to an intracellular shift of potassium because of thyrotoxicosis as no other cause for hypokalemia was found. Similarly she was also evaluated for myopathy, but no obvious cause was found apart from thyrotoxicosis.

The baseline investigations of the patient are shown in Table 1.

Considering her cholestatic pattern of jaundice she was started on tab propylthiouracil (PTU) at a dose of 50 mg twice daily and nonselective *β* blockers (propranolol at a dose of 40 mg daily) along with potassium supplementation to correct her hypokalemia. Gradually she improved clinically and biochemically and was able to walk after a period of 6 weeks and was discharged on 150 mg of PTU tab daily. She was under regular followup during which her thyroid profile normalized with almost near normalization of liver function test.

The follow-up investigations of the patient are shown in Table 2.

After about six months since her first admission, she was again admitted with worsening thyrotoxicosis and jaundice, proximal muscle weakness, and fever with chills for 5 days. This time on evaluation she had conjugated hyperbilirubinemia with raised AST/ALT (96 U/L and 61 U/L, resp.) and normal alkaline phosphatase (151 U/L) indicating hepatocellular injury along with a high total count suggestive of septicemia. No obvious cause for septicemia could be found even after extensive workup. The superimposed septicemia was thought to be the triggering factor for the worsening of her thyroid dysfunction that was initially well controlled with antithyroid drug.

Investigations of the patient at readmission are shown in Table 3.

PTU was stopped because of rise in liver enzymes (ALT and AST) keeping in mind the possibility of PTU induced hepatitis. Radioiodine therapy was considered but deferred due to financial constraints. She was started on lithium therapy (300 mg twice daily); *β* blocker was restarted (propranolol at a dose of 60 mg daily) with simultaneous administration of glucocorticoids (dexamethasone at a dose of 8 mg daily) to control her thyrotoxicosis. No obvious cause for septicemia could be found even after extensive workup. So broad spectrum antibiotic coverage was given empirically and changed periodically, but without any response. There was a progressive rise in ALT and AST levels without much change in alkaline phosphatase level even after stopping PTU. After about one week because of worsening thyrotoxicosis, lithium was stopped and methimazole at a dose of 10 mg daily was started. Hypokalemia was corrected using potassium supplementation as before, but this time she showed no improvement in her proximal muscle weakness. Rather, during hospital stay she had worsening of her muscle weakness with involvement of both proximal and distal muscle groups of lower limbs, which was predominantly a flaccid type of paralysis with generalized areflexia. There was also evidence of slight muscle atrophy. Neurology consultation was taken and she was diagnosed to develop a mixed axonal and demyelinating acute polyradiculoneuropathy. IV methylpredisolone therapy was started for treatment of this neuropathy. Meanwhile an upper GI endoscopy revealed extensive esophageal candidiasis for which IV antifungal treatment was also started. But in spite of all our efforts the patient succumbed to her illness.

## 3. Discussion

Clinically evident cholestasis with jaundice secondary to uncomplicated hyperthyroidism is rare. Elevated serum alkaline phosphatase is seen in 64% of patients with thyrotoxicosis [3]. However, this does not necessarily indicate that the liver is the source of origin, as it can originate from bone also. When jaundice occurs in a patient with thyrotoxicosis, multiple factors are believed to play a part in the pathogenesis, including heart failure, infection, and weight loss [4]. Once hyperthyroidism is controlled, cholestasis will improve. Although hyperthyroidism induced cholestatic injury of the liver is of mild degree, hyperthyroidism can be rarely complicated by a severe cholestatic syndrome that may dominate the clinical presentation and course [5]. In our case, features which indicate a primary role of hyperthyroidism per se in the pathogenesis of the cholestatic syndrome were the appearance of jaundice after the clinical onset of hyperthyroidism in this treatment naïve patient and its improvement with simultaneous improvement in thyroid dysfunction; the absence of congestive heart failure and of any infectious or toxic agents to liver; and the biochemical findings of cholestasis. Hull et al. in 2007 [6] reported two cases of severe jaundice (total bilirubin levels: 35.2 mg/dL and 42 mg/dL, resp.) associated with thyroid storm in the absence of intrinsic liver disease, thionamide exposure, or congestive heart failure who responded well to antithyroid drugs. Greenberger et al. [7] similarly reported four cases of jaundice and thyrotoxicosis in the absence of congestive heart failure.

Interestingly our patient presented with reappearance of jaundice at second admission, and this time the biochemical picture was suggestive of a hepatocellular injury rather than a cholestatic pattern as evident during the initial presentation. The exact cause of this hepatocellular damage could not be established even after an extensive workup. Although liver biopsy was not attempted in our case because of the general ill health of the patient, an initial negative screening test for anti-nuclear antibody test was presumably against the diagnosis of autoimmune hepatitis which may sometime occur in association with Grave's disease. Although we had planned for other autoimmune markers which are more specific for autoimmune liver injury like smooth muscle antibodies, antibodies to liver/kidney microsome type1, antimitochondrial antibodies, and perinulear antineutrophil cytoplasmic antibodies, the poor financial condition of the patient forced us to defer these costly investigations. In patients with chronic hepatitis associated with primary biliary cirrhosis (PBC) or chronic autoimmune hepatitis, there is an increased prevalence of autoimmune thyroid disease [8, 9]. Liver injury due to autoimmune hepatitis may be seen in as much as 10% of patients with hyperthyroidism due to Graves's disease [10, 11].

Our patient was effectively being controlled with a minimum dose of propylthiouracil (PTU) during her follow-up after the initial presentation. Increased serum levels of aspartate aminotransferase (AST) and alanine aminotransferase (ALT) may be present in about 30% of patients treated with propylthiouracil [12]. In the majority of patients, serum aminotransferases returned to normal following withdrawal of the drug. Rarely, a persistent hepatitis occurs with clinical, biochemical (elevated bilirubin, AST, and ALT), and histological features of hepatocellular necrosis [13]. It should be kept in mind that this antithyroid drug induced liver damage is an idiosyncratic reaction and does not depend on the dose and duration of exposure although it generally occurs during the first 3 months of starting of therapy. It may even occur despite previous uneventful exposure to the drug and involves less than 0.5% of patients [14]. PTU is the third most common cause of drug related liver failure and accounts for 10% of the drug related liver transplantation [15]. Ichiki et al. [16] reported a case of Grave's disease presenting with severe jaundice after starting PTU for two months and which completely resolved after stopping the drug with simultaneous use of IV methylprednisolone pulse therapy. A literature survey published in 1997 reported 49 cases of hepatotoxicity, 28 associated with PTU, including 7 deaths [12]. So the possibility of drug induced hepatitis should also be included in the differential diagnosis of the hepatocellular injury in our case.

The uncontrolled hepatocellular damage in our patient may also be a part of a multiorgan dysfunction (MODS) which occurred in association with septicemia due to an unknown cause [17]. Finally the possibility of hyperthyroidism per se leading to this predominantly hepatocellular dysfunction should be kept in mind although hyperthyroidism itself can cause only mild elevation in liver enzymes which normalizes with treatment and clinically apparent jaundice is rare in uncomplicated hyperthyroidism unless associated with heart failure and secondary liver dysfunction [7, 18]. The exact mechanism by which thyroid hormone causes hepatocyte injury is unclear. It may be because of direct toxic effects of thyroid hormone on the hepatocytes or of a relative hepatic ischemia secondary to peripheral vasodilatation leading to a mismatch between metabolic demand and supply [19, 20].

Our patient also developed an acute paraplegia with profound lower extremity weakness and areflexia at second admission during the course of her hospital stay. A nerve conduction study was suggestive of a mixed axonal and demyelinating polyneuropathy. The association of generalized peripheral neuropathy with thyrotoxicosis is rare. However, an acute neuropathy associated with paraplegia or quadriplegia has been described. The presentation has been described as Basedow's paraplegia by Joffroy in 1891 [21] and it resembles Guillain-Barre syndrome in terms of clinical presentation and nerve conduction studies. Symptoms and signs generally resolve with correction of thyrotoxicosis. This flaccid paralysis with absent reflexes and occasionally with some sensory deficit was reported several times by early European authors [22]. Ludin et al. in 1969 [23] described involvement of the peripheral nerves in hyperthyroidism by reporting eight patients with an electromyogram of distal leg muscles suggestive of “neurogenic” origin. Chollet et al. [24] also mentioned two cases of hyperthyroidism and polyneuropathy with definite slowing of nerve conduction in electrophysiological studies.

Since we cannot arrive at the exact etiology of hepatic dysfunction with our limiting resources and facilities, we try to treat the patient aiming at all possible mechanisms of liver injury in a patient with Grave's disease like stopping of the probable antithyroid drug causing hepatitis (PTU) and replacing it with other medications to control the thyrotoxicosis, namely, lithium, *β* blocker, and finally methimazole, using glucocorticoids to control thyrotoxicosis as well as to cover up the autoimmune probability of hepatitis and using broad spectrum antibiotics to treat the underlying infection. But we failed to offer a definitive therapy in the form of radioiodine ablation to treat her hyperthyroidism due to the financial barrier. Nonavailability of facilities for liver transplantation in our setup was also a major limiting factor in our treatment efforts. Finally failure to treat the systemic infection effectively even after using all available broad spectrum antibiotics and delayed use of antifungal therapy may have also been additive factors for our failure.

## 4. Conclusion

Hepatic dysfunction in a patient with thyrotoxicosis is not an uncommon presentation. But sometimes this simple biochemical abnormality may dominate the clinical picture and complicate the course of the primary disease. It is also evident that although multiple mechanisms may play their roles in development of hepatic dysfunction in a patient with thyrotoxicosis, it is sometimes difficult to establish definitely which factor is causing liver injury in a particular patient. It is probably impractical to separate and find all these causes out, and what seems more important is to focus on definitive treatment directed at saving both these two vital organs using a multidisciplinary approach. Presence of life threatening sepsis should always be carefully sought for in such clinical presentations and if present it should aggressively be treated. Sometimes it becomes very difficult to help such kind of patients even after our best possible efforts, particularly in a developing country like India where access to radioiodine ablation and liver transplantation may not be possible to all groups of patients. It is the need of time to formulate some specific guidelines for managing patients of thyrotoxicosis presenting with severe hepatic dysfunction with special emphasis on those situations where definitive therapy in the form of radioiodine ablation may not be feasible.

## Figures and Tables

**Table 1:** Baseline investigations of the patient.

TSH (mIU/L)	**0.056**	Serum alkaline phosphatase (SAP) (U/L) (38–126)	**364 **
FT4 (ng/dL) (0.89–1.7)	**3.45**	S. albumin (g/dL)	3.3
T3 (nmol/L) (1.25–2.74)	**4.56**	Prothrombin time (sec)	14.2
Anti-TPO Ab (IU/dL)	450	Serum CPK	Normal
Bilirubin (total) (mg/dL)	**15.8**	USG abdomen	Hepatomegaly with periportal cuffing suggestive of acute hepatitis
Conjugated (mg/dL)	**12.6**	Viral markers (HVB, HCV, HAV, and HEV)	Negative
Unconjugated (mg/dL)	1.5	Arterial blood gas analysis (ABG)	Normal
AST (U/L)	46	24-hour urinary potassium	Normal
ALT (U/L)	43	S. Na^+^/K^+^ (mmol/L)	134/2.7

**Table 2:** Follow-up investigations of the patient.

	At adm.	1st wk	2nd wk	3rd wk	At disch.	2nd month	5th month	6th month
TSH (mIU/L)	0.056		0.28	—			0.45	0.78
FT4 (ng/dL) (0.89–1.7)	**3.45 **				**2.1**	**1.9**	**1.69**	**1.24**
T3 (nmol/L)	4.56				3.34	3.3	2.67	2.01
S. bilirubin (Total) (mg/dL)	15.8	10.5	8.4	5.5	4.5	4.14	3.5	3.12
Conjugated (mg/dL)	**12.6 **	**8.4 **	**5.6 **	**3.4**	**3.1**	**2.89**	**1.6**	**1.45**
Unconjugated (mg/dL)	1.5	0.8	0.7	0.5	1.1	0.6	0.45	0.67
AST (U/L)	46	43	35	33	34	38	39	32
ALT (U/L)	43	44	37	32	36	40	36	34
Serum alkaline phosphatase (U/L)	**364 **	**252 **	**198 **	**168**	**155**	**133**	**101**	**98**
S. Na^+^ (mmol/L)	134	145	142	138	142	140	139	138
S. K^+^ (mmol/L)	2.7	3.8	4.1	4.3	4.0	3.9	4.1	3.9

**Table 3:** Investigations of the patient at readmission.

TSH (mIU/L)	<**0.001**	Urine R/E	2–6 pus cells/HPF, RBC-present
FT4 (ng/dL) (0.89–1.7)	**10.2** ng/dL	Urine culture	No growth on two occasions
T3 (nmol/L) (1.25–2.74)	>**9.24** nmol/L	Blood culture	No growth
TC (total count) (per cm)	**16250 **	X-ray chest PA view	Normal
DLC (differential leucocytic count)	Neutrophilia	2D echo	Normal
Erythrocyte sedimentation rate (ESR)	106 mm after the end of 1st hour	USG abdomen	Mild hepatomegaly, no evidence of chronic hepatitis, no evidence of cholangitis
C reactive protein (CRP) (0–10 mg/dL)	**58**		
S. bilirubin (Total) (mg/dL)	30.2		
Conjugated (mg/dL)	26.3	Malaria parasite	Negative
Unconjugated (mg/dL)	4.23	Widal test	Negative
AST (U/L)	**96 **	PBS study	No abnormal or immature cells seen
ALT (U/L)	**61 **	HBS Ag, anti-HCV Hepatitis A Hepatitis E-IGM	Negative
Serum alkaline phosphatase (U/L)	151	ANA	42 IU (weakly positive)
S. albumin (g/dL)	**2.3 **	S. ammonia	Normal
Prothrombin time (sec)	15.3		
S. Na^+^ (mmol/L)	138		
S. K^+^ (mmol/L)	3.1		
